# A New Class of Almost Ricci Solitons and Their Physical Interpretation

**DOI:** 10.1155/2016/4903520

**Published:** 2016-12-04

**Authors:** K. L. Duggal

**Affiliations:** Department of Mathematics and Statistics, University of Windsor, Windsor, ON, Canada N9B 3P4

## Abstract

We establish a link between a connection symmetry, called conformal collineation, and almost Ricci soliton (in particular Ricci soliton) in reducible Ricci symmetric semi-Riemannian manifolds. As a physical application, by investigating the kinematic and dynamic properties of almost Ricci soliton manifolds, we present a physical model of imperfect fluid spacetimes. This model gives a general relation between the physical quantities (*u*, *μ*, *p*, *α*, *η*, *σ*_*ij*_) of the matter tensor of the field equations and does not provide any exact solution. Therefore, we propose further study on finding exact solutions of our viscous fluid physical model for which it is required that the fluid velocity vector *u* be tilted. We also suggest two open problems.

## 1. Introduction

In general, there are two categories of geometric quantities and their corresponding two kinds of flows:* intrinsic* quantities, such as the metric of a manifold, and the* extrinsic* quantities such as the embedding of a manifold in some ambient space. The Ricci flow, the Calabi flow, and the Yamabe flow belong to the first category and the mean curvature flow (MCF) belongs to the second category. In 1982, Hamilton [1] introduced the concept of Ricci flow for Riemannian manifolds. The self-similar solutions to the Ricci flow (evolved purely by homotheties and diffeomorphisms) are called Ricci solitons. Since then his work has been used in resolving many longstanding open problems in Riemannian geometry and 3-dimensional topology. Basic details and a collection of research papers on this area of research are available in [2, 3], respectively. There are very limited papers on Ricci solitons for semi-Riemannian (in particular, Lorentzian) manifolds (e.g., see [4, 5]). On the other hand, the study on MCF is primarily focused on the fixed points of a submanifold of minimal volume embedded in some fixed space. Active research is going on all the above stated research areas on some aspects of geometry and physics, although most authors still prefer using Riemannian ambient space. Recently, Pigola et al. [6] have introduced a modified concept of the Ricci solitons equation (called* “almost Ricci solitons”*) by allowing the soliton constant *λ* to be a variable function Φ (see a brief account on this modification in Section 3). In this paper, we present a mathematical model of almost Ricci soliton (ARS) semi-Riemannian manifolds which also admit a connection symmetry, called* “conformal collineations*” (Definition 1) and a physical model of almost Ricci soliton imperfect fluid (in particular, viscous fluid) spacetimes of general relativity.

## 2. Conformal Collineations

The notion of symmetry is a very useful tool in geometry and physics. A comprehensive account on metric (i.e., Killing, homothetic, and conformal Killing) and connection (i.e., affine and conformal collineations) symmetries in semi-Riemannian (in particular, spacetime) manifolds can be found in [7] and references therein. In this paper we use the following* conformal collineation* symmetry introduced by Tashiro [8].

**Definition 1 .**

An (*n* + 1)-dimensional semi-Riemannian manifold $\left(\overset{-}{M},\overset{-}{g}\right)$ admits a conformal collineation symmetry defined by a vector field *V* if

(1) $$\begin{array}{c} {£}_{V}\Gamma_{ij}^{k}=\delta_{i}^{k}\Psi_{j}+\delta_{j}^{k}\Psi_{i}-{\overset{-}{g}}_{ij}\Psi^{k}, \end{array}$$

where Γ_*ij*_^*k*^ denotes Christoffel's symbols, Ψ is a function, and Ψ_*j*_ = ∂_*j*_(Ψ). *V* is then called an “*Affine Conformal Vector*” (ACV) field of $\overset{-}{M}$.

**Proposition 2 ([9, Sharma-Duggal (1985)]).**

A vector field *V* on a semi-Riemannian manifold $(\overset{-}{M},\overset{-}{g}$) is an ACV if and only if

(2) $$\begin{array}{c} {£}_{V}\overset{-}{g}=2\Psi \overset{-}{g}+K, \end{array}$$

where *K* is a (0,2) covariant constant $\left(\overset{-}{\nabla }K=0\right)$ symmetric tensor field.

An ACV reduces to a* conformal Killing vector*, briefly denoted by CKV, if *K* is proportional to $\overset{-}{g}$. Thus, an ACV deviates from a CKV field if there exists a second-order covariant constant symmetric tensor $K\neq \overset{-}{g}$. On the existence of an ACV and specific restrictions on the ambient manifold $\overset{-}{M}$, we recall that, in 1923, Eisenhart [10] proved that “*If a Riemannian manifold* $\overset{-}{M}$ *admits such a tensor K, independent of* $\overset{-}{g}$ *, then* $\overset{-}{M}$ *is reducible*.” This means that $\overset{-}{M}$ is locally a product manifold of the form ($\overset{-}{M}=M_{1}\times M_{2},\;\;\overset{-}{g}=g_{1}⊕g_{2}$) and there exists a local coordinate system in terms of which the distance element of $\overset{-}{g}$ is given by

(3) ds2=g−abxcdxadxb+g−ABxCdxAdxB;a,b,c=1,…,r≤n;  A,B,C=r+1,…,n+1.

Thus, an irreducible $\overset{-}{M}$ admits no ACV field. In 1951, Patterson [11] proved that “*If a semi-Riemannian* $\overset{-}{M}$ *admitting such a tensor K, independent of* $\overset{-}{g}$ *, is reducible then the matrix (K*_*ij*_ *) has at least two distinct characteristic roots at any point of* $\overset{-}{M}$.” Therefore, for a semi-Riemannian $\left(\overset{-}{M},\overset{-}{g}\right)$, a general characterization of an ACV still remains open. In 1925, Levy [12] proved that “*A second order covariant constant non-singular symmetric tensor in a space of constant curvature is proportional to the metric tensor.*” Thus, a semi-Riemannian manifold of constant curvature admits no ACV other than a CKV. Therefore, the study on ACV symmetry is restricted to only manifolds with nonconstant curvature. Physically, for example, Minkowski, de-Sitter or anti-de-Sitter spacetimes do not admit an ACV.

In particular, an ACV is called an* affine vector*, briefly denoted by AV, if Ψ = 0. Tashiro [8] has also proved that* “a globally defined ACV is necessarily an AV field.*" Therefore, for proper ACV our results in the paper will hold locally. For basic details (with examples) on ACV, see Tashiro [8], Patterson [11] Duggal [13], Mason-Maartens [14], and others referred therein.

## 3. Almost Ricci Soliton Semi-Riemannian Manifolds

Let $\left(\overset{-}{M},\overset{-}{g}\right)$ be an (*n* + 1)-dimensional semi-Riemannian manifold. Recall that the “*Ricci flow”* on $\overset{-}{M}$ is defined by the following equation:

(4) $$\begin{array}{c} \frac{\partial {\overset{-}{g}}_{ij}}{\partial t}=-2{\overset{-}{R}}_{ij}\;\left(i,j=1,\ldots ,n+1\right), \end{array}$$

where ${\overset{-}{R}}_{ij}$ is the Ricci tensor of $\overset{-}{M}$. A solution $\overset{-}{g}(t)$ of the Ricci flow equation (4) is called “*Ricci soliton”* if there exists a positive function *ϕ*(*t*) and a 1-parameter family of diffeomorphisms $\psi (t):\overset{-}{M}\to \overset{-}{M}$ such that $\overset{-}{g}(t)=\phi (t)\psi (t{)}^{*}\overset{-}{g}(0)$, where *ϕ*′(*t*) = −2*λ* for a constant *λ*. Substituting this data in the Ricci flow equation, we obtain

(5) $$\begin{array}{c} {\overset{-}{g}}_{ik}{\overset{-}{\nabla }}_{j}{\overset{-}{\nabla }}^{k}+{\overset{-}{g}}_{kj}{\overset{-}{\nabla }}_{i}{\overset{-}{\nabla }}^{k}={£}_{V}{\overset{-}{g}}_{ij}=2\lambda {\overset{-}{g}}_{ij}-2{\overset{-}{R}}_{ij}, \end{array}$$

where *V* is a vector field on $\overset{-}{M}$. We say that the vector field *V*, satisfying the evolution equation (5), is called a “*Ricci soliton vector,"* briefly denoted by RS vector, and $\left(\overset{-}{M},\overset{-}{g},\lambda ,V\right)$ is called a Ricci soliton (RS) manifold which is said to be shrinking, steady, or expanding if *λ* is positive, zero, or negative, respectively. The Ricci soliton manifolds are natural extension of Einstein manifolds and are self-similar (homothetic) solutions to their Ricci flow equation. In year 2011, Pigola et al. [6] introduced a modified class of the Ricci soliton equation (5) by replacing the soliton constant *λ* with a variable function Φ and then $\left(\overset{-}{M},\overset{-}{g},\Phi ,V\right)$ is called an “almost Ricci soliton” manifold, which we denote by ARS-manifold, and *V* the “*almost Ricci soliton vector,"* briefly denoted by ARS vector, such that the evolution equation (5) becomes

(6) $$\begin{array}{c} {£}_{V}{\overset{-}{g}}_{ij}=2\Phi {\overset{-}{g}}_{ij}-2{\overset{-}{R}}_{ij}. \end{array}$$

Similarly, we say that the ARS is shrinking, steady, or expanding if Φ is positive, zero, or negative, respectively. For an example of an ARS-manifold we refer to [15]. If *V* is the gradient of a smooth function *f*, up to the addition of a Killing vector field, then we can replace *V* by $\overset{-}{\nabla }f$ and $\left(\overset{-}{M},\overset{-}{g},\Phi ,V\right)$ is called a gradient ARS-manifold for which the evolution equation (6) assumes the form

(7) $$\begin{array}{c} {\overset{-}{\nabla }}_{i}{\overset{-}{\nabla }}_{j}f+{\overset{-}{R}}_{ij}=\Phi {\overset{-}{g}}_{ij}. \end{array}$$

Also, for the ARS solution of Ricci Flow, we consider

(8) $$\begin{array}{c} \overset{-}{g}\left(\;t\;\right)=\phi \left(\;t,x^{a}\;\right)\psi {\left(\;t\;\right)}^{*}\overset{-}{g}\left(\;0\;\right), \end{array}$$

where *ψ*(*t*) are diffeomorphisms of $\overset{-}{M}$ generated by a family of vector fields *X*(*t*) and *ϕ*(*t*, *x* ^*a*^) are pointwise scaling functions depending on all the coordinates (*t*, *x* ^*a*^) of points with the initial condition ${\overset{-}{g}}_{ij}(0)={\overset{-}{g}}_{ij}$, *ψ*(0) = *I* → *ϕ*(*t*, *x* ^*a*^) = 1. Differentiating (8) with respect to *t*, using the Ricci flow equation (4), we get

(9) $$\begin{array}{c} {\left(\;\frac{\partial }{\partial t}\phi \left(\;t,x^{a}\;\right)\;\right)}_{\left|t\right.=0}{\overset{-}{g}}_{ij}+{£}_{X\left(0\right)}{\overset{-}{g}}_{ij}=-2{\overset{-}{R}}_{ij}. \end{array}$$

Labelling *X*(0) = *V* and ((∂/∂*t*)*ϕ*(*t*, *x* ^*a*^))_|*t*=0_ = −2Φ we get the almost Ricci soliton equation (6).

For an ARS-manifold $\left(\overset{-}{M},\overset{-}{g},\Phi ,V\right)$, with dimension ≥ 3 and homothetic *V*, $\overset{-}{g}$ is Einstein for which Φ = *λ* so ARS-manifold is Ricci soliton. Also, for an ARS-manifold, the vector *V* is conformal if and only if $\overset{-}{g}$ is Einstein. So far we have references [6, 15–18] on ARS manifolds.

## 4. Ricci Symmetric Almost Ricci Soliton Manifolds

Recall that a Riemannian or semi-Riemannian manifold $\left(\overset{-}{M},\overset{-}{g}\right)$ is called Ricci recurrent if its Ricci tensor satisfies ${\overset{-}{\nabla }}_{X}\overset{-}{Ric}(Y,Z)=\alpha (X)\overset{-}{Ric}(Y,Z)$, where *α*(*X*) is a 1-form on $\overset{-}{M}$. In particular, $\overset{-}{M}$ is Ricci symmetric if $\overset{-}{\nabla }\;\overset{-}{Ric}=0$. For details we refer to [19]. Now we present a mathematical model of a class of Ricci symmetric semi-Riemannian manifolds $\left(\overset{-}{M},\overset{-}{g}\right)$ admitting an ACV vector field which also satisfies the evolution equation (6) of an ARS vector field. For this purpose, recall that in 1970 Katzin et al. [20] proved that a nonflat conformally flat manifold $\left(\overset{-}{M},\overset{-}{g}\right)$ admits an ACV whose covariant constant tensor *K* is given by

(10) $$\begin{array}{c} K=\Omega \overset{-}{Ric},\;\Omega =\text{nonzero function on }\overset{-}{M}, \end{array}$$

which also holds for a semi-Riemannian manifold (see Patterson [11]). This result restricts $\overset{-}{M}$ to Ricci recurrent $\left({\overset{-}{\nabla }}_{X}\overset{-}{Ric}(Y,Z)=-\partial (\log\Omega )(X)\overset{-}{Ric}(Y,Z)\right)$ spaces due to $\overset{-}{\nabla }K=0$. Thus, if we set *Ω* constant = −2 (i.e., take $\overset{-}{M}$ Ricci symmetric) and then substitute this value of *K* from (8) in (2) we obtain

(11) $$\begin{array}{c} {£}_{V}{\overset{-}{g}}_{ij}=2\Phi {\overset{-}{g}}_{ij}-2{\overset{-}{R}}_{ij}, \end{array}$$

which is exactly the ARS evolution equation (6). We also refer to Levine-Katzin [21] who have proved that “*A second order covariant constant symmetric tensor K in a conformally flat manifold is a linear combination of the metric tensor and the Ricci tensor.*” To relate this with the ARS evolution equation (6) we let $K_{ij}=a{\overset{-}{g}}_{ij}+b{\overset{-}{R}}_{ij}$ for some constants *a* and *b*. Taking *a* = 0 and *b* = −2 we recover (6). Moreover, Grycak [22] has proved that “*Levine-Katzin's result also holds for a conformally Ricci recurrent manifold with a locally exact recurrent form.*” For basic information on conformally recurrent manifolds, we refer to [23]. Thus, it is possible that a link of ACV symmetry with ARS may also hold for a variety of Ricci symmetric semi-Riemannian manifolds other than what we know from above references. For this reason, we state the following general result.

**Theorem 3 .**

Let $\left(\overset{-}{M},\overset{-}{g}\right)$ be an (*n* + 1)-dimensional locally reducible Ricci symmetric semi-Riemannian manifold of nonconstant curvature. Suppose $\overset{-}{M}$ admits an ACV field *V* defined by (2) such that its covariant constant tensor $K_{ij}=-2{\overset{-}{R}}_{ij}$. Then, *V* is also an ARS vector field. Therefore, $\left(\overset{-}{M},\overset{-}{g},\Phi ,V\right)$ is an ARS-manifold.

In general, an ARS vector field *V* admits following curvature identities (proof is similar to curvature identities on CKV [24]):

(12) £VR−ijkm=δimΦk;j+δjmΦi;k+Φ;img−jk+Φ;jmg−ik,£VR−ij=div⁡grad Φg−ij−n−1Φ;ij,£Vr−=2n div⁡grad Φ−2Φr−+2r−.

An ARS vector *V* is RS vector if Φ = *λ* a constant for which (12) reduces to

(13) £VR−ijkm=0=£VR−ij,£Vr−=21−λr−.

**Corollary 4 .**

Under the hypothesis of Theorem 3, (a) if *V* is an affine vector (AV) field, then $\overset{-}{M}$ is a steady RS-manifold. (b) If a Ricci soliton manifold $\left(\overset{-}{M},\overset{-}{g},\lambda \right)$ is Einstein (*n* ≥ 2)  $\left({\overset{-}{R}}_{ij}=(\overset{-}{r}/\left(n+1\right)){\overset{-}{g}}_{ij},\;\;\overset{-}{r}\neq 0\right)$, then V is Killing, $\overset{-}{r}=\dim\left(\overset{-}{M}\right)$, and $\left(\overset{-}{M},\overset{-}{g},\lambda \right)$ is a steady RS-manifold.

The case (a) holds since Φ = 0 if *V* is an AV. For case (b), using (13) and $\overset{-}{r}\neq 0$, we get

(14) £VR−ij=r−n+1£Vg−ij=0⟹£Vg−ij=0.

Therefore, *V* is Killing, $\overset{-}{r}=n+1=\dim\left(\overset{-}{M}\right)$, and $\overset{-}{M}$ is a steady RS-manifold.

**Remark 5 .**

Theorem 3 will certainly hold for above referred three classes of locally reducible Ricci symmetric semi-Riemannian manifolds. The reason for our choice of making general statement is to initiate research on finding more such cases for which Theorem 3 may hold.

## 5. Physical Interpretation

In support of Theorem 3, we now show the existence of physically meaningful solutions of a class of fluid spacetimes of general relativity. Let $\left(\overset{-}{M},\overset{-}{g},\Phi ,V\right)$ be a 4-dimensional spacetime manifold. Results will also hold for higher dimensions. The set of all integral curves given by a unit nonnull or null vector field *u* is called the congruence of nonnull or null curves. Here we consider timelike curves, also called flow lines. The acceleration of the flow lines along *u* is given by ${\overset{-}{\nabla }}_{u}u$. The projective tensor $h_{ij}={\overset{-}{g}}_{ij}+u_{i}u_{j}$ is used to project a tangent vector at a point *p* in $\overset{-}{M}$ into a spacelike vector orthogonal to *u* at *p*. The rate of change of the separation of flow lines from a timelike curve tangent to *u* is given by the expansion tensor *θ*_*ij*_ = *h*_*i*_^*k*^ *h*_*j*_^*m*^ *u*_(*k*;*m*)_. The expansion *θ*, the shear tensor *σ*_*ij*_, the vorticity tensor *ω*_*ij*_, and the vorticity vector *ω* ^*i*^ are

(15) θ=div ⁡u=θijhij,σij=θij−θ3hij,ωij=hikhjmuk;m,ωi=12ηijkmujωkm,

where *η* ^*ijkm*^ is the Levi-Civita volume-form. The covariant derivative of *u* satisfies

(16) $$\begin{array}{c} u_{a;b}=\omega_{ab}+\sigma_{ab}+\frac{\theta }{3}h_{ab}-u_{b}\left(\;u_{;a}^{c}u_{c}\;\right). \end{array}$$

The rate at which the expansion *θ* changes along *u* is given by Raychaudhuri equation as follows:

(17) $$\begin{array}{c} u\theta =\frac{d\theta }{ds}=-{\overset{-}{R}}_{ij}u^{i}u^{j}+2\omega^{2}-2\sigma^{2}-\frac{1}{3}\theta^{2}+\mathrm{div}\left(\;\nabla_{u}u\;\right), \end{array}$$

where *ω* ^2^ = (1/2)*ω*_*ij*_ *ω* ^*ij*^ and *σ* ^2^ = (1/2)*σ*_*ij*_ *σ* ^*ij*^ are nonnegative and *s* is an arc-length parameter.

### 5.1. Kinematics of ARS-Spacetimes

To find the kinematic properties of ARS-spacetimes satisfying (6) with unit fluid velocity vector *u*, we set *£*_*V*_ *u* ^*i*^ = *ϕu* ^*i*^ + *w* ^*i*^, for some functions *ϕ* and *w* ^*i*^, where *w* ^*i*^ *u*_*i*_ = 0. Contracting this with *u*_*i*_ and using ${£}_{V}u_{i}={£}_{V}({\overset{-}{g}}_{ij}u^{i})$, *£*_*V*_(*u*_*i*_ *u* ^*i*^) = 0, and the evolution equation (6) we obtain $\phi =u^{i}(2\Phi u_{i}-2{\overset{-}{R}}_{ij}u^{j}+{\overset{-}{g}}_{ij}{£}_{V}u^{j}).$ Therefore,

(18) $$\begin{array}{c} \phi u^{i}=-2\left(\;\Phi +{\overset{-}{R}}_{ij}u^{i}u^{j}\;\right)-{£}_{V}u^{i}. \end{array}$$

Substituting this value of *ϕu* ^*i*^ in *£*_*V*_ *u* ^*i*^ = *ϕu* ^*i*^ + *w* ^*i*^ we get

(19) £Vui=−Φ+R−jkujukui+wi2,£Vui=Φ−R−jkujukui−2R−ijui+wi2.

Decompose *V* ^*i*^ as *V* ^*i*^ = *αu* ^*i*^ + *β* ^*i*^, where *α* = −*u*_*i*_ *V* ^*i*^ and *β* ^*i*^ *u*_*i*_ = 0. Then, using (16) (see details given in [25]), we obtain ${£}_{V}u_{i}=\dot{\alpha }u_{i}+\alpha \left({\dot{u}}_{i}+(\ln\alpha^{-1}{)}_{;j}h_{i}^{j}\right)+\beta^{j}{\dot{u}}_{j}u_{i}+2\omega_{ij}\beta^{j}.$ Using this along with the second equation of (19) and contracting with *u* ^*i*^ and $h^{ik}={\overset{-}{g}}^{ik}+u^{i}u^{k}$ we get

(20) Φ=α˙+u˙iVi−R−ijuiuj,wi=2ωijVi+αu˙i+ln⁡α−1;jhij+2R−jkujhik.

With respect to a nonnull ARS vector field *V* we define the projection tensor $h_{ij}={\overset{-}{g}}_{ij}-\epsilon \alpha^{2}V_{i}V_{j}$ where *h*_*ij*_ *V* ^*i*^ = 0 and *V* · *V* = *ϵα* ^2^  (*ϵ* = ±1, *α* > 0).

**Proposition 6 .**

Let $\left(\overset{-}{M},\overset{-}{g},\Phi ,V\right)$ be a locally reducible Ricci symmetric ARS-spacetime which satisfies the evolution equation (6) with a nonnull ARS vector field *V*, where *V* · *V* = *ϵα* ^2^  (*ϵ* = ±1, *α* > 0). Then, the following kinematic relations hold:

(1) $\sigma_{ij}=\alpha^{-1}\left((1/3)h^{km}h_{ij}-h_{i}^{k}h_{j}^{m}\right){\overset{-}{R}}_{mk}$;
(2) $\theta =\alpha^{-1}\left(3\Phi -h^{ij}{\overset{-}{R}}_{ij}\right)$,

where *σ*_*ij*_ and *θ* are the shear tensor and the expansion of *V*.

The proof follows by contracting (6) with *h*_*i*_^*k*^ *h*_*j*_^*m*^ − (1/3)*h* ^*km*^ *h*_*ij*_ and *h* ^*ij*^, respectively. Also, it follows from Corollary 4(b) that, under the hypothesis of above proposition, an Einstein RS-spacetime $\left(\overset{-}{M},\overset{-}{g}\right)$ is expansion-free (*θ* ≡ 0) and shear-free (*σ*_*ij*_ ≡ 0).

**Remark 7 .**

We highlight that Proposition 6 will also hold for an RS-spacetime for which (Φ = *λ*). Therefore, it follows that the physical use of our class of ARS or RS-spacetimes $\left(\overset{-}{M},\overset{-}{g}\right)$ in general relativity has a role in the study of fluids with nonzero expansion and shear unless $\overset{-}{M}$ reduces to an Einstein RS-spacetime which is expansion-free and shear-free. It is important to mention that our choice of timelike ARS symmetry vector field *V* in this paper is due to the fact that the integral curves of such a timelike connection symmetry can provide a privileged class of observers or test particles in a spacetime. However, for a spacelike ARS symmetry vector *V* one can use the congruence of spacelike curves, introduced by Greenberg [26], and discuss its kinematic properties and work on its physical use similar to the results presented in next section.

### 5.2. Dynamics of ARS-Spacetimes

On the issue of any possible existence of physically meaningful solutions of our class of fluid ARS-spacetimes we must know how the Einstein field equations are related with purely kinematic results of previous subsection. For this purpose, consider the Einstein field equations

(21) $$\begin{array}{c} G_{ij}\equiv {\overset{-}{R}}_{ij}-\frac{1}{2}\overset{-}{r}\;{\overset{-}{g}}_{ij}=T_{ij},\;\overset{-}{r}=-T_{i}^{i}, \end{array}$$

where *G*_*ij*_ is the Einstein tensor. Suppose $\left(\overset{-}{M},\overset{-}{g}\right)$ is an ARS-spacetime with an ARS vector field *V* and satisfies the hypothesis of Theorem 3. It is known that the invariance (*£*_*V*_ *G* = 0) of *G* is physically desirable since that amounts to invariance of matter tensor *T*, useful in finding exact solutions. For example, we know that if a spacetime admits a Killing or homothetic symmetry vector *V*, then *V* leaves *G* (and therefore *T*) invariant, but this may not be true if spacetime admits some higher symmetry vector such as conformal Killing vector (CKV). However, we do know that there are some physically important exact solutions of spacetimes which admit CKV symmetry. For example, Maartens-Maharaj [27] have proved that Robertson-Walker spacetimes admit a 9-parameter group of CKVs. In the following we show that our case of the ARS or RS symmetry vector *V* is similar to the proper CKV symmetry. Indeed, assuming *£*_*V*_ *G* = 0 and using the curvature identities and the evolution equation (6) implies that *V* leaves *G* invariant only if there exists a spacetime which admits a covariant constant Ricci tensor of the form

(22) $$\begin{array}{c} {\overset{-}{R}}_{ij}={\overset{-}{g}}_{ij}+\frac{\left(n-1\right)}{\overset{-}{r}}\left[\;\mathrm{div}\left(\;grad\;\Phi \;\right){\overset{-}{g}}_{ij}+\Phi_{;ij}\;\right],\;\overset{-}{r}\neq 0. \end{array}$$

Thus, in general, the ARS or RS symmetry vector *V*, with $\overset{-}{r}\neq 0$, does not leave *G* invariant. We, therefore, propose the following open question:

*Does a spacetime with covariant constant Ricci tensor of the form (22) exist?*

In particular, if Φ = *λ*, then we know from Corollary 4(b) and (22) that $\overset{-}{M}$ is a steady Einstein manifold with Killing *V* and $\overset{-}{r}=\dim\left(\overset{-}{M}\right)=n+1.$

Now we look for physically meaningful solutions of a fluid ARS-spacetime $\left(\overset{-}{M},\overset{-}{g}\right)$. We first recall the following concept of* material curves* useful in the study of fluid spacetimes. A material curve in a fluid is a curve which moves with the fluid as the fluid evolves. Material curves play an important role in relativistic fluid dynamics. In our case we assume that the timelike ARS vector field *V* = *αu* means that *V* is a material curve as it maps flow lines into flow lines. Therefore, *w* ^*i*^ = 0 in two equations of (19). This also means that *u* is an eigenvector of ${\overset{-}{R}}_{ij}$ which does not hold in general. Now we recall from a paper of Hall-da Costa [28] that the existence of a covariant constant second-order tensor field, other than the metric tensor, must exclude some spacetimes, in particular, perfect fluids. Based on this result and our Remark 7, we choose an imperfect fluid matter tensor in following physical model.

**Theorem 8 .**

Let $\left(\overset{-}{M},\overset{-}{g},\Phi ,V\right)$ be a 4-dimensional locally reducible Ricci symmetric ARS-spacetime of nonconstant curvature with the evolution equation (6) for a timelike ARS vector *V* parallel to the fluid velocity vector *u*. Assume $\overset{-}{M}$ admits the following imperfect fluid Einstein field equations:

(23) R−ij−12r− g−ijTij=μ+puiuj+pg−+πij+qiuj+qjui,qiui0,πijuj0,

with *μ*, *p*, *q*, *π*_*ij*_, the density, thermodynamic pressure, energy vector, and the anisotropic pressure tensor, respectively. Then

(i) $\Phi =\dot{\alpha }-\left(\mu +3p\right)/2$, *V* = *αu*.
(ii) *π*_*ij*_ = −*ασ*_*ij*_, *q* ≠ 0.
(iii) If Φ = *λ* and $\overset{-}{r}$ is a nonzero constant, then $\overset{-}{M}$ is shrinking (*λ* = 1) RS-spacetime.

**Proof**

Using $\overset{-}{r}=-T_{i}^{i}=\mu -3p$ we get the following relations from the field equations (23):

(24a) R−ijuiuj=μ+3p2,

(24b) R−ijhkihmj=μ−phkm+πmk,

Since ARS vector *V* is timelike and parallel to *u* we let *V* = *αu*. This means that *V* maps flow lines into flow lines so *w* ^*i*^ = 0. Substituting *w* ^*i*^ = 0 in (19) and then using (24a) we get $\Phi =\dot{\alpha }-(\left(\mu +3p\right)/2)$ so (i) holds. Now using (24b) in item (1) of the Proposition 6 we get (ii). Finally, if Φ = *λ* and $\overset{-}{r}$ is nonzero constant, it follows from the second relation of (12) that *λ* = 1 so $\overset{-}{M}$ is a shrinking RS-spacetime so (iii) holds which completes the proof.

*Viscous Fluid*. A particular case of imperfect fluid is the viscous fluid with the viscosity coefficient *η*. It is known [29] that viscous fluid is characterized by the imperfect matter tensor (22) for which *π*_*ij*_ = −2*ησ*_*ij*_ such that *α* = 2*η* > 0. This establishes the relativistic equivalence to the classical Navier-Stokes theory of fluid mechanics [30]. Thus, *V* = 2*ηu*. Consequently, by adjusting the magnitude of the ARS vector *V*, one can measure the response of the viscous fluid subject to the amount of viscosity fluctuations of the fluid.

## 6. Discussion

The motivation for this work comes from the use of a connection symmetry (Definition 1) in the study of semi-Riemannian almost Ricci soliton (ARS) manifolds $\left(\overset{-}{M},\overset{-}{g},\Phi ,V\right)$, recently introduced by Pigola et al. [6]. The idea originated from close similarity of (2) of the affine conformal vector (ACV) field of connection symmetry with the ARS vector filed *V* of the evolution equation (6) of the ARS manifolds as stated in Theorem 3. We highlight that use of some basic results on ACV symmetry from references [7, 13, 14] in Section 5, under the condition that $\overset{-}{M}$ is reducible and Ricci symmetric, plays a key role in finding some physically meaningful solutions of imperfect Einstein field equations, in particular viscous fluids. Also, it is clear from Proposition 6 that as the fluid revolves parallel to the velocity vector *u* the presence of shear causes distortion in the fluid and this change in the fluid pattern is governed by the deviation of the Ricci tensor ${\overset{-}{R}}_{ij}$ from the metric tensor $\overset{-}{g}$. A stage may come when the ARS-spacetime $\overset{-}{M}$ is Einstein which it is sheer-free and then it follows from (22) that ${\overset{-}{R}}_{ij}={\overset{-}{g}}_{ij}$. On the other hand, for the case of ARS-spacetime (${\overset{-}{R}}_{ij}\neq {\overset{-}{g}}_{ij}$), one can adjust the magnitude of the ARS vector filed *V* in response of viscous fluid subject to large viscosity fluctuations in order to describe the leading finite-size correction in scaling needed for a problem under investigation. However, our physical Theorem 8 only gives a general relation between the quantities (*u*, *μ*, *p*, *α*, *η*, *σ*_*ij*_) and does not provide any exact solution. The reason is that the exact solutions of the field equations for a viscous fluid require tilting velocity vector and it cannot be determined by the field equations alone. One needs to specify the type of physical model under investigation. For example, Coley and Tupper [29] have used tilted velocity vector in presenting radiation-like viscous fluid exact solutions for a prescribed FRW model. In another paper we will follow the method used in [29] and study some more works on viscous fluids for completing our goal of finding physically acceptable exact solutions based on the results of this paper. Finally, we propose following problem.

*Open Problem*. It has been shown in [31] that locally conformally flat Lorentzian gradient Ricci solitons are locally isomorphic to a Robertson-Walker warped product, if the gradient of the potential function is nonnull, and a plane wave, if the gradient of the potential function is null, but for this later case gradient Ricci solitons are necessarily steady. We leave it as an open problem to further study in reference [31], satisfying the hypothesis of our Theorem 3.
